# *In Vitro* Antioxidant Activities of Sulfated Derivatives of Polysaccharides Extracted from *Auricularia auricular*

**DOI:** 10.3390/ijms12053288

**Published:** 2011-05-18

**Authors:** Hua Zhang, Zhen-Yu Wang, Lin Yang, Xin Yang, Xue Wang, Zhi Zhang

**Affiliations:** 1 School of Food Science and Engineering, Harbin Institute of Technology, 73 HuangHe Road, NanGang District, Harbin 150090, China; E-Mails: zhhua@hit.edu.cn (H.Z.); ly6617@hit.edu.cn (L.Y.); yangxin@hit.edu.cn (X.Y.); 2 School of Forestry, Northeast Forestry University, 26 HeXing Road, DongLi District, Harbin 150040, China; E-Mail: ldzhangzhi@163.com; 3 Symrise (Shanghai) Co., Ltd., Shanghai 201206, China; E-Mail: 774234134@qq.com

**Keywords:** polysaccharides, *Auricularia auricular*, sulfated derivatives, antioxidant activities, *in vitro*

## Abstract

In this research, two types of sulfated polysaccharide derivatives were successfully synthesized. Their antioxidant activities were investigated by employing various established *in vitro* systems. In addition, the degree of sulfation was evaluated using ion-chromatography and IR spectra. The results verify that, when employing scavenging superoxide radical tests, both the sulfation of acid *Auricularia auricular* polysaccharides (SAAAP) and the sulfation of neutral *Auricularia auricular* polysaccharides (SNAAP) derivatives possessed considerable antioxidant activity and had a more powerful antioxidant competence than that of the native non-sulfated polysaccharides (AAAP and NAAP). On the other hand, AAAP and NAAP exhibited stronger activity on scavenging both the hydroxyl radical and lipid peroxidation. Available data obtained with *in vitro* measurements indicates that the sulfated groups of AAAP and NAAP played an important role on antioxidant activity. In sum, the research demonstrates that the antioxidant activity of sulfated polysaccharide derivatives *in vitro* has a potential significance for seeking new natural antioxidant protective agents.

## 1. Introduction

The aim of this study is to improve the antioxidant activities of polysaccharides from Auricularia Auricura by sulfation. Reactive oxygen species (ROS) in the forms of superoxide anion (•O_2_^−^), hydroxyl radical (•OH) and hydrogen peroxide (H_2_O_2_) are charged and highly reactive species that are made of unstable molecules or atoms due to their single and unbalanced electrons, and they can attack vital biomolecules, such as DNA, lipids, and proteins in cells and body fluids [1]. In cellular oxidation reactions, the superoxide radical is primarily formed where its properties can be magnified as it produces other kinds of cell-damaging free radicals and oxidizing agents. The uncontrolled generation of free radicals is associated with lipid and protein peroxidation resulting in cell structural damage, tissue injury, or gene mutation and ultimately leads to the development of various health disorders such as Alzheimer’s disease, cancer, atherosclerosis, diabetes mellitus, hypertension, and ageing [2].

For that reason, searching and identifying natural and safe antioxidants to protect the human body from various sources of oxidative damage, especially of plant origin, has notably increased in recent years [3,4]. The intake of dietary antioxidants may help to maintain an adequate antioxidant status in the human body [5]. In particular, polysaccharides have been demonstrated to play an important role as a dietary free radical scavenger in the prevention of oxidative damage in living organisms [6,7].

There are several methods to measure the efficiency of dietary antioxidants either as pure compounds or in food matrices. These methods focus on the different mechanisms of the antioxidant defense system, such as: the scavenging of oxygen and hydroxyl radicals, the reduction of lipid peroxyl radicals, the inhibition of lipid peroxidation, or the chelation of metal ions [8,9]. The interaction between free radicals (such as superoxide and hydroxyl radicals) and antioxidants can show direct evidence for antioxidants to scavenge free radicals and has been widely used to evaluate the radical scavenging ability of antioxidants [5].

Many studies have also demonstrated that chemical modifications can improve water solubility of poor water-soluble fractions and provide an opportunity to obtain new pharmacological agents with possible therapeutic uses [10,11]. Many scientific researchers have been exploring this potential [12–15].

The *Auricularia* mushroom species is the fourth most important cultivated mushroom used by humans throughout the world [16]. The fruit of *A. auricula-judae* is rich in hetero-polysaccharides that consists of a backbone d-glucose residue with various chains of β-1,3-branch residues, such as mannose, glucose, xylose and glucuronic acid. Moreover, many published studies have examined the beneficial health benefit from *A. auricula-judae* on both humans and animals. Of significance, *A. auricula-judae* is known for its pharmaceutical effects, such as: inhibiting lipid peroxidation, decreasing liver damage [17,18], antioxidant activity, scavenging radicals, chelating metal ions *in vitro* [18]; anti-oxidative and hypolipidemic properties [19]. Research has also shown it has a beneficial pharmacological effect on the left ventricle ejection fraction in aged mice [20].

This paper demonstrates that by using Cetyl Trimethyl Ammonium Bromide (CTAB) the *A. auricula-judae* water-soluble polysaccharide could first be separated into two parts—neutral and acidic. Following a further necessitous modification of the neutral and acidic polysaccharide by sulfated modification, the resultant was analyzed by Fourier Transform Infrared Spectroscopy (FTIR) and High-performance Gel-permeation Chromatography (HPGPC). Finally, a systematically and potentially beneficial health effect due to antioxidant activity *in vitro of* the sulfated derivatives of polysaccharides extracted from *A. auricula-judae*, was then investigated.

## 2. Materials and Methods

### 2.1. Samples

The fruit body of *A. auricula-judae* was purchased from a Carrefour supermarket in Harbin, Heilongjiang Province, China.

### 2.2. Reagents

The T-series Dextrans were obtained from Agilent, Beijing, China. Nicotinamide adenine dinucleotide-reduced (NADH), Nitro blue tetrazolium (NBT), Phenazine methosulphate (PMS), Deoxyribose, 2,2′-Azinobis-(3-ethylbenzthiazoline-6-sulphonate) (ABTS) were purchased from Sigma Chemicals Co. (St. Louis, MO, USA). FeSO_4_, EDTA, hydrogen peroxide (H_2_O_2_), sodium borohydride, chlorosulphonic acid (HClSO_3_), *N*,*N*-dimethyl formamide (DMF), formamide (FA), Cetyl Trimethyl Ammonium Bromide (CTAB), Tris, HCl, 2-thiobarbituric acid (TBA), trichloroacetic acid (TCA) were of analytic reagent grade and made in China. All solutions were made in double-distilled water.

### 2.3. Extraction of *A. auricula-judae* Polysaccharide

*A. auricula-judae* polysaccharide (AAP) was extracted by hot water and ultrasonic-assisted extraction. The concentrated supernatants were then precipitated with three volumes of absolute ethanol (95%) and maintained at 4 °C overnight. The resulting precipitate was separated by centrifugation, dissolved in deionized water, and then dialyzed. The non-dialyzed portion was, in addition, lyophilized to give a crude polysaccharide extract. The AAP was then decolorized by hydrogen peroxide (H_2_O_2_); this process was critical to the manufacture of a fine polysaccharide product.

### 2.4. Fraction of *A. auricula-judae* Polysaccharide

The separation of acidic (AAAP) and neutral polysaccharides (NAAP) was performed using Cetyl Trimethyl Ammonium Bromide (CTAB) or Cetylpyridinium Chloride (CPC) as this forms a precipitated complex with the acidic polysaccharide. The fine polysaccharide allows for a further purification treatment by the CTAB fractionation process [21].

### 2.5. Sulfation of AAAP and NAAP

Modified slightly, the sulfation of AAP was prepared according to the method defined by Wang *et al.* [22]. The sulfation reagent, SO_3_-DMF, was obtained by dropping 50 mL HClSO_3_ into 300 mL DMF and cooled in an ice-water bath. Dry AAAP and NAAP (1.0 g) was added to 40 mL FA, and the mixture was stirred at 50 °C for 3 h in order to disperse into solvent. Then 10 mL SO_3_-DMF reagent was added. After 3 h, the mixture was cooled to room temperature and precipitated with 75% ethanol for 24 h. Following this procedure, the precipitate was washed three times with 60% ethanol, and then dissolved in 100 mL of distilled water. The solution was neutralized with 1 mol/L NaOH solution and dialyzed against tap water for 48 h and distilled water for 24 h using 3500 Da Mw cutoff dialysis membrane.

### 2.6. Determination of Degree of Sulfating (DS)

Sulfated AAAP and NAAP in a tube with a screw cap was hydrolyzed by adding 4 mL 1 mol/L HCl at 100 °C for 6 h, respectively. Hydrolysis liquid was volatilized with a nitrogen stream at 45 °C. The sulfate group contents of two sulfated *Auricularia auricular* polysaccharides were determined by ion-chromatography. A calibration curve was drawn taking sodium sulfate as standard. The degree of sulfating (DS) was calculated according to the equation: DS = (1.62 × S%)/(32 − 1.02S%).

### 2.7. Amino Acid Analysis

Amino acids in SAAAP and SNAAP were determined with a Hitachi L8800 automatic amino acid analyzer (Hitachi Ltd., Tokyo, Japan). The hydrolysis of the samples was done in a sealed ampoule for 24 h at 110 °C using 1 mL of 6 mol/L HCl solution under vacuum. The hydrolysate was evaporated and then the dried residue dissolved in 0.02 mol/L HCl. Finally, the sample was filtered through a 0.45 μm nylon filter before being injected into the amino acid analyzer.

### 2.8. Analytical High-Performance Liquid Chromatography (HPLC)

The molecular weight (M_w_) of SAAAP and SNAAP was measured by High performance Gel-Permeation Chromatography (HPGPC) on an Agilent 1100 HPLC system (Agilent, USA) equipped with a differential refractive index detector (RID). As well, an automatic sample injector was used. All separations were performed with a PL Aquagel-OH Mixed column (300 mm × 7.5 mm i.d. × 8 mm—Agilent, USA) and then the retention times were plotted against the logarithms of their corresponding average molecular weights. All spectra were acquired by ChemStation software system (Agilent, USA). The mobile was 0.02% NaN_3_ water solution and with a flow-rate of 1.0 mL/min. A 50 μL sample passed through a 0.45 μm cellulose acetate filter was injected in each run. The molecular weight was estimated by reference to the calibration curve made from a Dextran T-series standard of known molecular weight (106 Da, 194 Da, 620 Da, 1470 Da, 4120 Da, 11,840 Da, 25,820 Da, 58,400 Da, 124,700 Da, 465,000 Da, 965,000 Da, and 1250,450 Da).

### 2.9. Infrared Spectroscopy Analysis

Infrared (IR) analyses of the sulfated *A. auricula-judae* polysaccharides were obtained by grinding a mixture of polysaccharide with dry KBr and then pressing it into a mould. The IR spectra were recorded on a Spectrum one FT-IR spectrometer (PerkinElmer, USA) run in the 4000–400 cm^−1^ region.

### 2.10. Superoxide Anion Scavenging Activity

Measurement of superoxide anion scavenging activity was based on the method described by Nishikimi *et al.* (1972) with slight modification [23]. The superoxide radicals were generated in the PMS–NADH system and containing 1.0 mL Tris–HCl buffer (16 mmol/L, pH 8.0), 0.5 mL NADH (78 μmol/L), 0.6 mL NBT (50 μmol/L), 0.2 mL PMS (45 μmol/L) and a 0.2 mL samples at varying concentrations (0.2–10.0 mg/mL).

The mixture prepared earlier, was incubated at room temperature for 5 min and the absorbance read at 560 nm against the blank. The control was substituted with a Tris-HCl buffer. Decreased absorbance of the reaction mixture indicated increased superoxide anion scavenging activity. The capability of scavenging to superoxide radical was calculated using the following equation:

Scavenging effect %=(1−[A560]sample/[A560]control)×100

### 2.11. Hydroxyl Radical Scavenging Activity

The hydroxyl radical scavenging activity of the sample was measured using a modified Smirnoff and Cumbes’ method [24]. The hydroxyl radical (OH^−^) was generated from the Fe^2+^ ascorbate-EDTA-H_2_O_2_ system (Fenton’s reaction), which attacks the deoxyribose and sets off a series of reactions that ultimately result in the formation of MDA which can be measured as a pink MDA-TBA chromogen at 532 nm [25]. The reaction mixture contained 0.2 mL of different samples (0.2–5.0 mg/mL) and was incubated in a dark room with 0.8 mL FeSO_4_ (0.4 mmol/L), 0.2 mL EDTA (1 mmol/L), 0.2 mL H_2_O_2_ (10 mmol/L) and 0.2 mL deoxyribose (15 mmol/L) for 30 min at 37 °C. In addition, 1.4 mL TBA-TCA-HCl (TBA-TCA reagent: 0.375% (w/v) 2-thiobarbituric acid (TBA), 15% (w/v) trichloroacetic acid (TCA) and 0.25 N HCl) was added and heated at 100 °C for 15 min. Following the cooling of the mixture to room temperature, the hydroxyl radical was detected by monitoring absorbance at 532 nm. The control sample was then substituted with distilled water and replaced the H_2_O_2_. The capability of scavenging to the hydroxyl radical was calculated using the following equation:

Scavenging effect %=(1−[A532]sample/[A532]control)×100

### 2.12. ABTS Radical Scavenging Assay

The ABTS radical scavenging activity of the extracts from *A.auricular* was measured according to the method of Ajandouz *et al.* (2001) with slight modification [26]. The working solution was prepared by mixing 7.4 mmol/L ABTS solution and 2.6 mmol/L potassium persulfate solution in equal quantities, allowing this amalgam to react for 12 h at 4 °C in a dark environment. It was then diluted by mixing the ABTS solution with methanol in order to obtain an absorbance of 0.700 ± 0.02 units at 734 nm using a T-6 spectrophotometer (PUXI apparatus company, China). A fresh ABTS solution was then prepared for each assay. A 150 μL of the sample (0.2–10.0 mg/mL) was mixed with 2850 μL of the ABTS solution. The mixture was then vigorously shaken by hand and left to stand for 2 h in the dark, the absorbance was then measured at 734 nm.

The scavenging ability was calculated as follows:

Scavenging effect %=(1−[A734]sample/[A734]control)×100

### 2.13. Assay of Lipid Peroxidation

The antioxidative effects of the extracts from *A.auricula* on liposome-induced oxidation with FeCl_3_-ascorbate and lipid peroxidation were quantified based on a MDA production by the method described by Duh (1999), with slight modification [27]; through liposomes that were prepared from 100 mg lecithin mixed with 10 mL of sodium phosphate buffer (10 mmol/L, pH 7.4). When shaken vigorously in a cold bath, this mix resulted in a final concentration of 10 mg/mL liposomes. The reaction mixture containing 1.0 mL of liposome, 1.0 mL FeSO_4_ (0.4 mmol/L) and 1.0 mL different samples (0.2–5.0 mg/mL); to make sure that the mixture was incubated for 60 min at 37 °C in dark room. In addition, 2.0 mL TBA-TCA-HCl (TBA-TCA reagent: 0.375% (w/v) TBA, 15% (w/v) TCA, and 0.25 N HCl) was added and heated at 100 °C for 15 min. Following cooling of the mixture to room temperature, the absorbance of the ensuing mix was detected by monitoring absorbance at 532 nm. The scavenging ability was calculated as follows:

Scavenging effect %=(1−[A532]sample/[A532]control)×100

## 3. Results and Discussion

### 3.1. Preparation

Mushroom polysaccharides exist as a structural component of a fungal cell wall. Selection of an extraction method depends on the cell wall structure. In the past, hot water extraction has been a popular approach. Mizuno (1996) developed reliable procedures for successful extraction of polysaccharides from fruiting bodies, or cultured mycelia [28].

In this research, the yield of *A. auricula-judae* polysaccharide extracted by hot water was 11.33%. Yang and Zhang (2009) found the separation of an acidic and a neutral polysaccharide is possible using Cetyl Trimethyl Ammonium Bromide (CTAB), where the outcome is a precipitated complex with the acidic polysaccharide [29]. In this research, when the *A. auricula-judae* polysaccharide was twice the volume of CTAB, the precipitation reached a maximum of 0.2174 g. In addition, when using CTAB, the polysaccharide was twice the volume of CTAB and the reaction time of 3.5 h was the optimal parameter to obtain maximum acidic *A. auricula-judae* polysaccharide [21].

### 3.2. Determination of DS

Two sulfated derivatives were prepared with CSA–DMF method. The resultant solution in the dialysis membrane was concentrated and lyophilized to give sulfated SNAAP and SAAAP, washed three times with 60% ethanol, and then dried at 95 °C (mean yield 51.89% and 85.03%). The result showed that the sulfate group contents of two AAAP and NAAP were not detected by ion-chromatography. The sulfate group contents of sulfated AAAP and NAAP were determined by ion-chromatography. It was concluded that the DS of SAAAP and SNAAP were 0.90 and 0.78, respectively, in accordance with the sulfate group content and that the sulfate group content of the sulfated AAAP and NAAP was higher than that of native non-sulfated polysaccharide (AAAP and NAAP).

### 3.3. Amino Acid Analysis by Amino Acid Analyzer

During the analysis of the polysaccharide sample, the protein was removed to avoid subsequent interference in determining the Mw of the polysaccharides. In spite of this, in this research, neither the Sevag method [30], nor the application of the proteinase method in removing protein, was adopted. The total amino acid composition in the SAAAP and SNAAP was determined by using an amino acid after the SAAAP and the SNAAP were hydrolyzed. Consequently, the total amino acid content indicates the protein content.

Table 1 shows the content of the SAAAP and SNAAP acquired acquired by CTAB; and includes the sulfated modification. From this Table, the proteins of the *A. auricula-judae* polysaccharide fraction did not exhibit high content.

### 3.4. Molecular Mass Distribution

**Figure 1.**
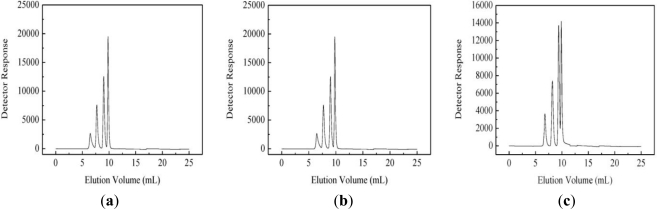

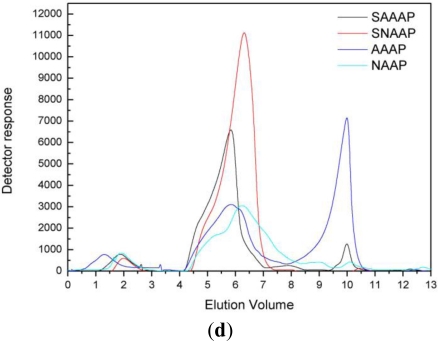
Gel-permeation Chromatography (GPC) of standard dextran (**a**–**c**), and sample (AAAP, SNAAP, SAAAP and SNAAP) (**d**).

A typical size-exclusion GPC chromatogram of SAAAP and SNAAP gave a peak region of 4–10 min (Figure 1). The retention time of SAAAP and SNAAP was then plotted on the same graph and weight-average molecular weight of the SAAAP and SNAAP were 8.09 × 10^6^ and 6.53 × 10^6^, when referenced to a standard dextran. Moreover, molecular mass distribution of sulfated AAAP and NAAP were narrower than that of native non-sulfated polysaccharide (AAAP and NAAP). This shows that sulfated AAAP and NAAP were two homogeneous samples.

### 3.5. Fourier Transform Infrared (FT-IR) Spectroscopy Analysis

**Figure 2.**
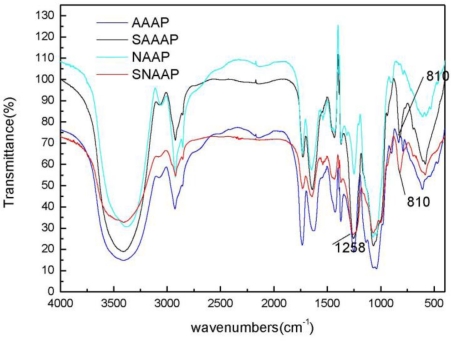
Fourier Transform Infrared (FTIR) spectra of AAAP, SAAAP, NAAP, and SNAAP.

An FT-IR spectroscopy analysis was used to investigate the vibrations of molecules and polar bonds between the different atoms. It is possible to analyze structures of polysaccharides such as monosaccharide types, glucosidic bonds, and functional groups using an FT-IR spectroscopy [29]. The SAAAP and SNAAP in the frequency range of 4000–400 cm^−1^ were then pulverized and blended with a KBr powder, which was then pressed into pellets for the FT-IR measurement (Figure 2). In all spectra, the band in the region of 3411.1 and 3410.5 cm^−1^ of SAAAP and SNAAP corresponds to the hydroxyl stretching vibration of the polysaccharide and at 2923.3 and 2921.3 cm^−1^ corresponds to a weak C–H stretching vibration. This amalgamation indicates that the SAAAP and SNAAP were all polysaccharides. By comparison with AAAP and NAAP, two characteristic absorption bands appeared in the FT-IR spectra of the sulfated derivatives (Figure 2): One at 1258 cm^−1^ describing an asymmetrical S=O stretching vibration and the other at 810 cm^−1^ representing a symmetrical C–O–S vibration associated with a C–O–SO_3_ group, which signifies that they were successfully sulfated [31].

### 3.6. Scavenging Activity of Superoxide Radical Analysis

Due to their antioxidant components, the consumption of certain natural products are now widely associated with the chemoprevention of degenerative diseases such as cancer and cardiovascular disorders. In particular, polysaccharides have been demonstrated to play an important role as a dietary free radical scavenger, especially in the prevention of oxidative damage in living organism [32–34]. As for *A. auricula-judae* polysaccharide, using experimental mice, researchers have found it had anti-tumor immunomodulation, anti-mutagenic and protected the mitochondria of the liver and brain of the experimental mice [20,35–37].

**Figure 3.**
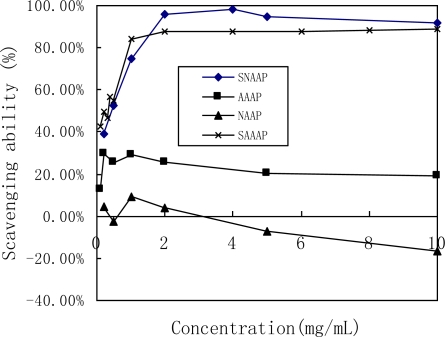
Scavenging effect on superoxide radicals by AAAP, SAAAP, NAAP and SNAAP.

The scavenging ability of all tested samples (including AAAP, SAAAP, NAAP, and SNAAP) on the superoxide radicals showed significance in a concentration-dependent manner at low concentration (Figure 3). Efficient concentration was defined as the concentration that inhibited a 50% radical generation or a scavenging 50% radical. This concentration, in turn, generated EC_50_ of sulfated AAAP and sulfated NAAP of 0.27 and 0.20 mg/mL, respectively.

It was found that the scavenging ability of Vitamin C against the superoxide radical was only 68.19% at 2.0 mg/mL [38]. The scavenging ability of the superoxide radical by all tested sulfated samples was more than 85% at a concentration greater than 2.0 mg/mL.Therefore, SAAAP and SNAAP had a much stronger scavenging activity on the superoxide radical than Vitamin C and the native non-sulfated polysaccharide (AAAP and NAAP). Tsiapali *et al.* (2001) found that phosphated and sulfated glucan showed greater antioxidant ability. This finding indicated the polyelectrolytes, such as glucan sulfate, or phosphate, may increase scavenging activity [6]. The superoxide anion is one of the precursors of the singlet oxygen and hydroxyl radicals, therefore it indirectly initiates lipid peroxidation. In addition, the presence of superoxide anion can magnify the cellular damage as it produces other kinds of free radicals and oxidizing agents to induce pathological events such as arthritis and Alzheimer’s disease [39]. Therefore, direct scavenging of superoxide radical has been a model for determining antioxidant activity [40]. These results clearly showed that the antioxidant activity of all sulfated samples were related to the ability of scavenging superoxide radicals.

### 3.7. Scavenging Activity of Hydroxyl Radical Analysis

**Figure 4.**
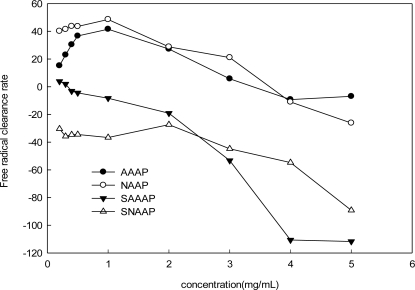
Scavenging effect on hydroxyl radicals by AAAP, SAAAP, NAAP, and SNAAP.

The hydroxyl radicals, generated by the Fenton reaction, were scavenged by AAAP, NAAP and its sulfated derivatives (Figure 4). Not all the sulfated derivatives exhibited stronger scavenging ability against hydroxyl radicals than the native non-sulfated polysaccharide did (AAAP and NAAP). For example, the activities of AAAP and NAAP increased the range from 0.2 to 1.0 mg/mL, while the activities (y of AAAP and NAAP) decreased when the concentration increased from 1.0 to 5.0 mg/mL range. The sulfated derivatives ranging from 0.2 to 5.0 mg/mL did not exhibit any scavenging ability against the hydroxyl radicals. The EC_50_ of these polysaccharides could not be read.

Previous studies have reported two types of antioxidant mechanism: suppression against hydroxyl radical generation and cleaning the hydroxyl radical generated [41]. The former mechanism is related to the transition of metal ions. In the absence of transition, metal ions, such as hydrogen peroxide, become relatively stable. However, hydroxyl radicals, when brought about by super-oxidation, and when engendered by hydrogen peroxide with metal ions, such as ferrous or copper, together with the amino and hydroxyl groups in the polysaccharide molecule; and then, when chelated with the metal ions, become influenced by the acidity of the solution. The pH of sulfated *Auricularia auricular* polysaccharide derivatives, when dissolved in such a solution, was not suitable for amino and hydroxyl groups to chelate ferric ion. Therefore, the activity of the sulfated *Auricularia auricular* polysaccharides for scavenging ability, as against hydroxyl radicals, was not as good as that of the native non-sulfated *Auricularia auricular* polysaccharides.

### 3.8. ABTS Radical Scavenging Assay

**Figure 5.**
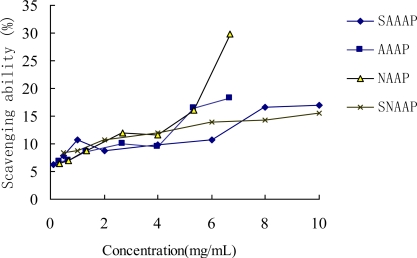
ABTS radical scavenging activity by AAAP, SAAAP, NAAP, and SNAAP.

The reactions with ABTS+• radicals involve a single-electron transfer process. Bleaching of a preformed solution of the blue-green radical cation ABTS+•, which has an absorption at 734 nm, has been extensively used by past researchers to evaluate the antioxidant capacity of complex mixtures and individual compounds [5]. As illustrated in Figure 5, the ABTS radical-scavenging activities of polysaccharides (including SAAAP, AAAP, SNAAP and NAAP) increased in four patterns with increased concentrations. A fast increase for native non-sulfated polysaccharide (NAAP and AAAP) and a slow increase for sulfated polysaccharides ranged from 0.1 to 6.67 mg/mL. The EC_50_ of these polysaccharides was also unreadable. The results also indicate that native non-sulfated *Auricularia auricular* polysaccharides and their sulfated derivatives are effective as natural antioxidants.

### 3.9. Assay of Lipid Peroxidation

**Figure 6.**
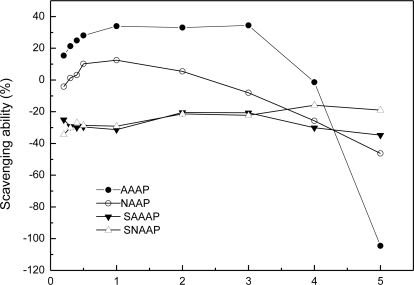
Lipid peroxidation by AAAP, SAAAP, NAAP, and SNAAP.

Cellular damage is closely related with lipid peroxidation and causes aging, carcinogenesis and many other diseases [42]. Liposomes are used extensively as biological models for *in vitro* lipid peroxidation studies [27,43]. Therefore, in this research the liposomal system was employed to evaluate the inhibitory activities of the *A.auricular* polysaccharide extracts toward lipid peroxidation. The results are noteworthy when compared to the findings of other studies concerning medicinal plants [44]. The study of lipid peroxidation is now increasing due to interest in finding natural herbal plants that can prevent not only lipid peroxidation in foods, but also oxidative damage to living cells. As illustrated in Figure 6, the lipid peroxidation inhibitory effect of polysaccharides (including AAAP and NAAP) increased in two patterns with increasing concentrations, while the scavenging activity of AAAP and NAAP decreased when the concentration range increased from 1.0 to 5.0 mg/mL. The sulfated *Auricularia auricular* polysaccharide derivatives, when tested over 0.2–5.0 mg/mL, did not show scavenging activity against lipid peroxidation. The EC_50_ of these polysaccharides was unreadable. Previous studies have reported that the antioxidant mechanism was the same as the cleaning hydroxyl radical when generated [5]. Therefore, the tendency of inhibitory activity against lipid peroxidation of *Auricularia auricular* polysaccharides (including SAAAP, SNAAP, AAAP, and NAAP) was consistent with the scavenging ability against the hydroxyl radicals.

## 4. Conclusions

This research supports that polysaccharides extracted from mushrooms have significant therapeutic potential and represent a rich source for future discovery and the development of novel compounds of medical value. The results clearly demonstrated two *A. auricular* derivatives: sulfated neutral polysaccharides and acid polysaccharides were synthesized successfully and that the average DS of sulfated AAAP and NAAP was 0.90 and 0.78, respectively. Both of the sulfated derivatives (range from 0.2 to 10.0 mg/mL) exhibited stronger antioxidant activity than *A. auricular* polysaccharides in superoxide radical scavenging activity assays *in vitro*. However, they didn’t exhibit stronger activity on scavenging hydroxyl radical, ABTS, and lipid peroxidation in contrast with native non-sulfated AAAP and NAAP.

The purpose of this study was to explore and provide an opportunity to obtain new or strong pharmacological agents with possible therapeutic use by means of chemical modifications. Sulfating can improve water solubility of poorly water-soluble fractions such as SAAAP and SNAAP. The findings conclude the need for further study to elucidate the relationship between structural features of sulfated polysaccharide fractions and their bioactivities other than antioxidant activities.

## Figures and Tables

**Table 1.** Amino Acids Content of SAAAP and SNAAP (%).

**Amino Acids**	**SAAAP**	**SNAAP**	**Amino Acids**	**SAAAP**	**SNAAP**	
Cys	0.78	1.42	Ile	not detected	not detected
Met	not detected	not detected	Leu	0.28	0.39
Asp	0.25	0.32	Tyr	0.25	0.35
Thr	0.19	0.25	Phe	not detected	not detected
Ser	0.19	0.26	Lys	not detected	not detected
Glu	0.41	0.57	His	0.08	0.10
Gly	0.09	0.12	Arg	not detected	not detected
Ala	0.19	0.23	Pro	not detected	not detected
Val	0.16	not detected	total amino acids	2.87	4.01
